# Comparing Quality of Life and Well-Being Among Patients with Chronic Diseases

**DOI:** 10.3390/healthcare14182923

**Published:** 2026-09-09

**Authors:** Mohamad Adam Bujang, Wei Hong Lai, Xun Ting Tiong, Sing Yee Khoo, Fazalena Johari, Alex Ren Jye Kim, Yvonne Yih Huan Jee, Clare Hui Hong Tan, Nurul Fatma Diyana Ahmad

**Affiliations:** 1 Clinical Research Centre, National Institutes of Health, Sarawak General Hospital, Ministry of Health Malaysia, Kuching 93586, Malaysia; laiweihong@crc.moh.gov.my (W.H.L.); tiongxt.crc@gmail.com (X.T.T.); khoosingyee@gmail.com (S.Y.K.); zazy2182@yahoo.com (F.J.); 2 Sarawak General Hospital, Ministry of Health Malaysia, Kuching 93586, Malaysia; alexkimsgh@gmail.com (A.R.J.K.); yvonnejee91@hotmail.com (Y.Y.H.J.); clare.tan.hui.hong@gmail.com (C.H.H.T.); 3 Sarawak Heart Center, Ministry of Health Malaysia, Kuching 94300, Malaysia; nurulfatmadiyana@gmail.com

**Keywords:** chronic diseases, health-related quality of life, quality of life, SigQOLM, well-being

## Abstract

**Highlights:**

**What are the main findings?**

- Health-related issues among patients with chronic diseases affects non-health-related aspects.
- In a stable condition (stable here refers only to care setting rather than clinical severity), patients with depressive disorders experience poorer quality of life and well-being compared with patients with other chronic diseases.

**What are the implications of the main findings?**

- It becomes necessary to use a generic scale to compare quality of life and well-being across different groups of patients with chronic diseases when comparisons across different diseases are required.
- Medical practitioners and policymakers can use these findings to design and tailor interventions aimed at improving the quality of life and well-being of patients with chronic diseases.

**Abstract:**

**Background**: Chronic diseases are widely recognized to negatively affect individuals’ quality of life and well-being. Therefore, this study aimed to evaluate the quality of life and well-being among patients with chronic diseases. **Methods**: This cross-sectional study using a self-administered survey was conducted from May 2022 to May 2023 in Sarawak General Hospital, Kuching, Malaysia. Data were collected based on a consecutive sampling technique from patients diagnosed with chronic diseases, including end-stage renal disease, cancer, heart disease, and depressive disorder. During the recruitment process, all patients were in stable condition (stable here refers only to care setting rather than clinical severity) and were recruited from four specialist clinics. All participants completed the Significant Quality of Life Measure (SigQOLM), a comprehensive instrument comprising 69 items that assess 18 domains and four dimensions of quality of life and well-being. Descriptive analysis and General Linear Model Analysis of Covariance (ANCOVA) were applied in the analysis. **Results**: A total of 168 patients were included in the study. Of these, 41 (24.4%) had end-stage renal disease (ESRD), 48 (28.6%) had cancer, 39 (23.2%) had heart disease, and the remaining 40 (23.8%) had depressive disorder. Statistical analysis demonstrated significant differences (*p* < 0.001) in quality of life and well-being among the four chronic disease groups. Despite being in stable condition, patients with depressive disorder reported the poorest quality of life and well-being, whereas patients with cancer and heart disease demonstrated the highest quality of life and well-being (adjusted mean score of 71.6 and 69.6, respectively). **Conclusions**: Under stable condition, differences in quality of life and well-being were observed among patients with chronic diseases. These findings provide valuable insights for medical practitioners and policymakers in designing and tailoring interventions to improve the quality of life and well-being of patients with chronic diseases.

## 1. Introduction

It is widely acknowledged that chronic diseases significantly affect individuals’ health-related quality of life and well-being [1,2,3]. Disease-specific quality of life instruments are particularly useful for evaluating symptoms and treatment outcomes within a specific condition because they are more responsive to disease-related changes [4]. However, these instruments are generally unsuitable for direct comparisons across different diseases because they measure different constructs using different domains and scoring systems. Consequently, generic quality of life instruments has been recommended when comparisons across disease groups are required. Some studies further emphasized that many generic instruments primarily assess health-related quality of life rather than the broader concept of overall quality of life [5,6]. Therefore, generic quality of life instruments are important for facilitating comparisons across different chronic diseases [7]. Nevertheless, there remains a scarcity of studies directly comparing quality of life and well-being among patients with different chronic diseases using a recently developed multidimensional generic instrument. One possible reason is that many researchers have developed disease-specific instruments to measure quality of life and well-being for particular conditions [8,9,10]. As a result, direct comparisons across different chronic diseases are often difficult. Such comparisons are more feasible when a generic instrument is used.

Quality of life (QOL), health-related quality of life (HRQOL), and well-being are closely related but conceptually distinct constructs. QOL is a broad multidimensional concept reflecting an individual’s overall perception of life, encompassing physical, psychological, social, and other life domains, whereas HRQOL focuses specifically on the aspects of quality of life influenced by health and disease [5,6]. Well-being represents a broader positive state that includes individuals’ perceptions of happiness, life satisfaction, and functioning. Various quality of life (QOL) and well-being instruments have been developed. However, most of these scales were introduced more than two decades ago [10,11,12,13]. One recently developed instrument is the Significant Quality of Life Measure (SigQOLM), introduced in 2023. The SigQOLM consists of 69 items covering four elements and 18 domains. The four elements include health, relationships, functional activities, and survival. In other words, an individual is considered to have an excellent quality of life and well-being when they experience positive health, meaningful relationships, satisfactory functional activities, and a favorable sense of survival and life continuity [14,15].

Therefore, this study provides an opportunity to explore disparities in quality of life and well-being using a recently developed, reliable, and validated instrument. Although generic quality of life instruments have been widely used for comparisons across disease groups, comparing multiple chronic diseases using a recently developed multidimensional instrument that encompasses health, relationships, functional activities, and survival remains limited. Accordingly, the present study utilized the SigQOLM to assess the quality of life and well-being among patients with four major chronic diseases, namely end-stage renal disease (ESRD), cancer, depressive disorders, and heart disease. The objective of this study was to compare the quality of life and well-being of these patient groups while they were in stable condition.

## 2. Materials and Methods

### 2.1. Study Design and Data Collection

This cross-sectional study and self-administered survey aimed to evaluate the QOL and well-being of patients with four different chronic diseases using the SigQOLM, Malaysia. Data collection was conducted from May 2022 to May 2023 using a consecutive sampling technique.

### 2.2. Patient Characteristics

Patients were recruited from four specialist clinics, namely nephrology (ESRD), oncology (various cancers), cardiology (heart disease), and psychiatry (depressive disorders) clinics. The inclusion criteria were: (i) patients currently receiving follow-up care at the respective specialist clinics, (ii) aged 18 years and above, and (iii) respondents (patients) only one severe chronic disease, either ESRD, cancer, heart disease, or depression. Individuals who were unconscious, comatose, or clinically or mentally unstable such that they were unable to provide informed consent or complete the self-administered questionnaire were excluded from the study. Data collection was collected during scheduled follow-up visits at specialist outpatient clinics and not during hospitalization or emergency care. In addition, all participants were willing to participate in the study and provided informed consent prior to completing the questionnaire.

### 2.3. Data Collection

Participants were recruited using consecutive sampling from four specialist clinics. All patients attending the respective clinics during the study period were screened for eligibility. Patients who fulfilled the predefined inclusion and exclusion criteria were invited to participate, and recruitment continued consecutively until the required sample size was achieved. No surveys from the recruited patients were withdrawn or omitted from the analysis.

### 2.4. Instrument

The SigQOLM is a multidimensional instrument developed to assess overall quality of life (QOL) and well-being across several key elements and domains that reflect important aspects of an individual’s life. The instrument comprises four main elements: Health (9 domains), Relationship (3 domains), Functional Activities (3 domains), and Survival (3 domains). The SigQOLM uses a standardized scoring approach in which responses to questionnaire items are transformed into scores ranging from 0 to 100, with higher scores indicating better perceived quality of life and well-being [13]. Based on the scoring mechanism, the Health element carries a greater weight in the overall SigQOLM score compared with the other three elements. Currently, the SigQOLM is available in two versions: the original version containing 69 items and a shorter version consisting of 36 items. In the present study, the full 69-item version of the SigQOLM was used [13,16].

### 2.5. Ethical and Regulatory Considerations

Only individuals who provided informed consent were included in the study. The confidentiality and privacy of participants were maintained throughout the study. Personal identifiers were removed or coded to protect the participants’ identities, and the data were used solely for research purposes in accordance with applicable data protection requirements. The study was conducted in accordance with all relevant guidelines and regulations established by the Medical Research and Ethics Committee (MREC) of the National Institutes of Health, Ministry of Health Malaysia. Ethical approval was obtained from the Medical Research and Ethics Committee (MREC) under NMRR ID-21-01979-XDL (IIR). 

### 2.6. Sample Size

The data for the primary study objective was analyzed using General Linear Model (GLM) Analysis of Covariance (ANCOVA). Accordingly, the sample size calculation was performed based on the ANCOVA sample size formula using PASS 2020 Power Analysis and Sample Size software (NCSS, LLC. Kaysville, Utah, USA). The revised sample size statement was as follows: “The aim of this study is to determine whether an independent variable (X1) is associated with an outcome (Y) after adjusting for four other covariates. The coefficient of determination (R^2^) attributable to X1 (R^2^T) was assumed to be 0.10, while the combined R^2^ of the four covariates (R^2^C) was also assumed to be 0.10. Based on these assumptions, a minimum sample size of 65 participants is required to detect a significant association between X1 and Y using a two-sided test with a significance level (α) of 0.05 and a statistical power of 80%.”. After accounting for an anticipated 20% non-response rate, the required sample size was increased to 82 participants [17].

### 2.7. Statistical Analysis

Descriptive analysis was performed to describe the participants’ profiles and to compare the QOL and well-being of patients with the four chronic diseases. The multivariate analysis used General Linear Model Analysis of Covariance (ANCOVA) to compare QOL and well-being domains across the different chronic disease groups. The dependent variables were the overall score for the SigQOLM and the four elements of quality of life and well-being (i.e., Health, Relationship, Functional activities, and Survival). The independent variable was the four chronic diseases as the main exposure. Meanwhile, gender, age group, ethnicities, and highest education level were controlled in the analysis. The post hoc analysis was performed based on the Bonferroni test. Associations with a *p* < 0.05 and sizeable effect size (i.e., partial eta-squared > 0.13) were considered significant to promote transparency in reporting [18,19]. The assumptions of the GLM/ANCOVA were assessed and found to be satisfied. These included homogeneity of variances (Levene’s test, *p* > 0.05), absence of multicollinearity (VIF < 2.0), approximately normally distributed residuals, and an adequate model fit, as indicated by a random scatter of residuals against predicted values. All statistical analyses were performed using SPSS software (IBM SPSS Statistics for Windows, version 20.0, released 2011, IBM Corp., Armonk, NY, USA).

## 3. Results

All analyses had an *n* = 168 and no missing data was observed. The study population comprised 168 patients, including 41 (24.4%) diagnosed with end-stage renal disease (ESRD), 48 (28.6%) with cancer, 39 (23.2%) with heart disease, and 40 (23.8%) with a depressive disorder. The majority of participants were female (61.3%), aged between 18 and 35 years (31.0%), and of Malay ethnicity (44.0%) (refer to Table 1). Although the minimum sample size was estimated to be 65 participants based on the ANCOVA power calculation, recruitment was continued beyond this minimum target, resulting in a final sample of 168 participants. This approach was adopted because sample size calculations are based on assumed parameters, which may differ from the characteristics observed in the actual study population. Recruiting a larger sample therefore provided greater assurance against potential underestimation of the required sample size and increased the precision and robustness of the statistical analyses. The larger sample was also considered appropriate given that several independent variables were included as covariates in the ANCOVA models.

### 3.1. Status of QOL and Well-Being Among Patients with Chronic Diseases

Although all patients with chronic diseases were in stable condition, they generally experienced moderate levels of health, with all groups reporting mean scores below 75.0% (third quartile). Among the four groups, patients with depressive disorder reported the poorest quality of life and well-being based on overall score and scores for the four elements, whereas patients with cancer and heart disease demonstrated the highest quality of life and well-being scores. Patients with cancer, ESRD, and heart disease reported excellent relationships and functional activities, with mean scores above 75.0%. In contrast, patients with depressive disorder reported mean scores below 75.0% across the remaining elements (Table 2).

### 3.2. QOL and Well-Being Among the Four Groups of Chronic Patients

Table 2 presents the mean scores for each SigQOLM domain according to diagnosis. All patient groups experienced low to moderate levels (mean scores below 75.0%) in domains such as pain, physical energy, sleep quality, eating regime (the pattern of food intake and dietary practices adopted by an individual that influence overall quality of life), and perception of future health. Patients with depressive disorder reported the poorest scores, particularly for sleep quality (32.0%), followed by psychological symptoms (33.1%), physical energy (40.9%), pain (41.9%), and perception of future health (43.9%). Despite being in stable condition, patients with cancer reported the highest overall quality of life and well-being scores, followed by patients with heart disease, ESRD, and depressive disorder (Table 2).

Quality of life and well-being differed significantly among patients with chronic diseases. The results were statistically significant (*p* < 0.001) with sizeable effect sizes (i.e., partial eta-squared > 0.13). Across all four elements and the overall quality of life and well-being score, patients with depressive disorders reported significantly lower scores than those with end-stage renal disease (ESRD), cancer, and heart disease (all *p* < 0.05). In contrast, no significant differences were observed in the scores among patients with ESRD, cancer, and heart disease (*p* > 0.05) (Table 3). The primary variable of interest was the type of chronic disease; therefore, the main emphasis was placed on the adjusted association between disease type and quality of life and well-being. For this reason, the results for the confounding variables are not presented in Table 3. Nevertheless, none of the confounding variables showed a statistically significant association with quality of life and well-being after adjustment (*p* > 0.05).

## 4. Discussion

Chronic diseases are well recognized to affect not only physical health but also psychological, social, and functional aspects of life, thereby influencing overall quality of life and well-being [20,21,22,23,24]. In this study, the use of a unified measurement tool across different disease groups facilitates direct comparison of quality of life and well-being across distinct chronic conditions. Nevertheless, these comparisons should be interpreted cautiously and are contingent on the instrument demonstrating adequate validity across the disease groups. This offers meaningful evidence to guide clinicians and policymakers in developing interventions that address patient needs in a more holistic and targeted manner.

The results indicate that even among patients who were in stable condition (refers only to care setting rather than clinical severity), meaningful differences in quality of life and well-being were still evident. Patients with end-stage renal disease, cancer, and heart disease generally demonstrated relatively favorable outcomes in non-health domains, including relationships, functional abilities, and perceptions of survival, with scores typically falling within the good to excellent range. Nevertheless, the health domain remained comparatively lower, suggesting that physical limitations persist despite overall clinical stability. One possible explanation is that patients may have partially adapted to the psychosocial and functional aspects of their lives, even though physical health challenges continue to affect their overall well-being [25,26].

Based on descriptive observation, patients with depressive disorder consistently showed the lowest scores across all domains of quality of life and well-being except for the mobility domain and eating regime domain. The impairment was not confined to health alone but extended across relationships, functional activities, and survival. This finding indicates that depressive disorder was associated with poorer quality-of-life scores across multiple dimensions reflecting its broad relationship with different aspects of life. Compared with patients with other chronic illnesses in a stable condition, patients with depressive disorders reported poorer quality of life and well-being across multiple dimensions.

These findings suggest that individuals with depressive disorders may benefit from careful psychosocial assessment in addition to routine clinical management. Although this study did not evaluate interventions or treatment effects, the findings highlight the potential importance of considering social support, coping strategies, daily functioning, and emotional well-being when planning comprehensive patient care. These clinical implications should be interpreted as preliminary and warrant further investigation [27]. In contrast, patients with physical chronic diseases may benefit more from interventions focused on physical health management while maintaining their relatively better preserved psychosocial functioning.

The differences in quality of life observed across the disease groups should also be considered in the context of differences in clinical characteristics that may accompany each chronic condition. Although the present analysis adjusted for gender, age group, ethnicity, and highest education level, unfortunately, detailed clinical characteristics were not assessed. These factors may influence quality of life independently of the disease category and may differ substantially between patients with ESRD, cancer, heart disease, and depressive disorders. Consequently, the observed between-group differences may reflect a combination of the underlying disease and its associated clinical burden. The absence of these clinical variables makes it difficult to determine the extent to which the observed differences are attributable to the disease category itself. Therefore, the findings should be interpreted as differences in quality of life between the studied disease groups after adjustment for selected sociodemographic characteristics, rather than as evidence that disease type alone determines quality of life.

An important concern with regard to the conceptual overlap between some SigQOLM domains and symptoms associated with major depressive disorder. In particular, psychological symptoms, sleep quality, physical energy, and aspects of eating behavior may be influenced by depressive symptomatology. Consequently, the lower SigQOLM scores observed among patients with depressive disorders may partly reflect shared symptom content rather than a completely independent reduction in quality of life. Although the SigQOLM was designed to assess broader perceived quality of life rather than to diagnose depression, this overlap may introduce a degree of criterion contamination and should be considered when interpreting the differences between disease groups. Therefore, the findings should not be interpreted as demonstrating that all of the observed difference in quality of life is independent of depressive symptomatology. Future research should assess the extent of this overlap through sensitivity analyses excluding potentially overlapping domains and/or by examining the relationship between SigQOLM scores and validated measures of depression severity.

Future research should consider longitudinal designs to examine how quality of life and well-being evolve over time, particularly in relation to disease progression and treatment response. Such studies would provide valuable insights into trajectories of change and critical periods for intervention. Further work should also expand the application of the SigQOLM across different populations to strengthen its generalizability and utility as a universal measure of quality of life and well-being.

Several limitations should be considered when interpreting the findings. First, the cross-sectional design precludes any causal inference regarding the association between chronic disease type and quality of life and well-being. Second, participants were recruited from specialist clinics within a single setting, and all were in a stable clinical condition. “Stable” here refers only to care setting rather than clinical severity. Therefore, the findings may not be generalizable to patients with more severe, unstable, or rapidly deteriorating conditions, or to other healthcare settings. Third, although statistically significant differences were observed between disease groups, the sample size within each group was relatively modest, which may have limited the precision of the estimates and the ability to detect smaller between-group differences. In addition, the disease groups were broadly categorized, and important clinical characteristics, including disease severity and other relevant clinical factors, were not collected. Although several demographic variables were adjusted for in the analyses, the possibility of residual confounding cannot be excluded.

The authors acknowledge that independent validation of the SigQOLM beyond studies conducted by the team that developed it is not yet available. Therefore, further independent validation in other countries is warranted. A further limitation is that, the number of eligible patients who declined participation was not systematically recorded. Thus, a refusal rate could not be calculated, and the possibility of selection bias cannot be excluded. Finally, access to the de-identified data is subject to approval by the Director General of the Ministry of Health Malaysia, which may limit independent reanalysis and replication of the findings.

## 5. Conclusions

This study identified differences in quality of life and well-being among patients with different chronic disease conditions. Patients with depressive disorders reported poorer outcomes across multiple domains compared with other chronic disease groups, even when in stable clinical condition. These findings highlight the importance of considering psychosocial factors in the assessment and management of chronic illnesses and may contribute to future research on integrated and patient-centered approaches to improve quality of life and well-being.

## Figures and Tables

**Table 1:** Demographic profile of patients.

Parameter	Characteristic	*n*	%
Gender	Male	65	38.7
	Female	103	61.3
Age Group	18–35	52	31.0
	36–40	23	13.7
	41–50	32	19.0
	50–60	40	23.8
	>60	21	12.5
Ethnicity	Malay	74	44.0
	Chinese	35	20.8
	Iban	28	16.7
	Bidayuh	22	13.1
	Melanau	2	1.2
	Others	7	4.2
Group	ESRD	41	24.4
	Cancer	48	28.6
	Heart	39	23.2
	Depressive	40	23.8

**Table 2:** Mean score with standard deviation of each SigQOLM domain of patients with various chronic diseases.

Domain/Element	ESRD		Cancer		Depressive		Heart	
	Mean	SD	Mean	SD	Mean	SD	Mean	SD
Pain	50.2	18.3	55.3	21.0	41.9	24.1	53.2	22.4
Physical energy	43.8	29.7	45.4	22.1	40.9	28.6	56.1	22.5
Psychological symptoms	72.6	25.5	78.7	23.8	33.1	23.1	79.3	18.9
Independence	80.5	25.1	89.4	16.8	70.6	29.2	84.0	20.0
Mobility	78.4	23.2	81.7	19.9	87.5	20.8	78.2	22.6
Sleep	59.9	29.1	62.8	21.6	32.0	24.2	65.2	18.8
Eating regime	27.7	29.1	38.4	30.8	56.3	23.7	47.0	27.4
Body image	70.4	25.5	75.3	27.3	40.2	32.6	68.9	28.9
Percep. of health	50.2	33.1	60.8	30.3	43.9	27.3	62.2	22.0
*Health*	59.9	16.6	66.4	15.0	49.0	16.4	65.7	14.3
Relationship with family	90.4	15.0	90.8	18.7	74.4	22.9	86.6	14.3
Relationship with religion	88.4	18.2	88.8	16.0	70.5	26.7	83.5	16.5
Relationship with friends	86.2	17.0	91.5	12.4	70.9	22.1	88.5	13.2
*Relationship*	88.2	13.9	90.4	10.9	72.3	16.3	86.1	12.2
Social activities	78.4	18.3	80.2	18.3	50.9	19.1	81.9	15.4
Time usage	83.0	14.1	81.2	18.0	56.8	23.5	81.2	16.5
Self-care activities	84.1	11.6	86.0	11.9	65.0	17.0	84.2	13.1
*Functional activities*	88.7	13.9	94.6	8.1	82.0	19.6	89.5	15.2
Safety	89.9	14.3	94.0	10.8	75.1	21.7	83.8	17.3
Basic needs	85.5	18.4	88.9	15.9	73.5	20.7	86.1	16.3
Percep. of future conditions	72.6	20.3	82.8	19.4	55.3	24.0	83.9	15.3
*Survival*	82.4	13.0	88.9	11.9	67.7	18.8	84.5	14.5
*SigQOLM*	67.1	10.9	71.7	10.4	54.9	13.8	69.4	10.5

Note: The name of the four elements and overall score were written in italic. Percep. Refers to perception.

**Table 3:** Comparisons of quality of life and well-being in four chronic diseases.

Outcome	*p*-Value (ES)	Adj. Mean	95% CI		Differences with
*Element*					
Health	<0.001 (0.138)				
a. ESRD		60.6	54.7	66.4	-
b. Cancer		68.0	61.9	74.1	-
c. Depressive		47.4	39.6	55.2	a, b, and d
d. Heart disease		66.8	61.0	72.6	-
Relationship	<0.001 (0.185)				
a. ESRD		88.8	83.7	93.8	-
b. Cancer		87.9	83.0	92.7	-
c. Depressive		69.6	62.9	76.4	a, b, and d
d. Heart disease		85.4	80.5	90.2	-
Functional activities	<0.001 (0.197)				
a. ESRD		84.3	79.1	89.5	-
b. Cancer		86.3	81.3	91.4	-
c. Depressive		64.6	57.7	71.6	a, b, and d
d. Heart disease		84.2	79.1	89.3	-
Survival	<0.001 (0.162)				
a. ESRD		84.3	78.9	89.8	-
b. Cancer		88.0	82.7	93.3	-
c. Depressive		66.9	59.7	74.2	a, b, and d
d. Heart disease		84.5	79.1	89.9	-
*Overall*					
SigQOLM	<0.001 (0.172)				
a. ESRD		67.7	63.1	72.2	-
b. Cancer		71.6	66.8	76.4	-
c. Depressive		53.4	47.0	59.9	a, b, and d
d. Heart disease		69.6	65.2	74.0	-

Note: Adj: Adjusted. ES: Refers to effect size represent by partial eta squared. CI: Confidence interval. The analyses were performed after adjusting for gender, age group, ethnicity, and highest education level.

## Data Availability

Data are available from the corresponding author upon approval from the Director General of the Ministry of Health Malaysia. The de-identified data can be requested from the corresponding author.

## Abbreviations

The following abbreviations are used in this manuscript:

ESRD	End-stage renal disease
MREC	Medical Research and Ethics Committee
SigQOLM	Significant Quality of Life Measure
QOL	Quality of life
WHOQOL BREF	A shortened version of the WHOQOL-100 developed by the World Health Organization Quality of Life Group

## References

[1] Mokhtari-Hessari P., Montazeri A. (2020). Health-related quality of life in breast cancer patients: Review of reviews from 2008 to 2018. Health Qual. Life Outcomes.

[2] Alwhaibi M., AlRuthia Y., Sales I. (2023). The impact of depression and anxiety on adult cancer patients’ health-related quality of life. J. Clin. Med..

[3] Nagy E., Tharwat S., Elsayed A.M., Shabaka S.A.E., Nassar M.K. (2023). Anxiety and depression in maintenance hemodialysis patients: Prevalence and their effects on health-related quality of life. Int. Urol. Nephrol..

[4] Guyatt G.H., Feeny D.H., Patrick D.L. (1993). Measuring health-related quality of life. Ann. Intern Med..

[5] Karimi M., Brazier J. (2016). Health, health-related quality of life, and quality of life: What is the difference?. PharmacoEconomics.

[6] Hand C. (2016). Measuring health-related quality of life in adults with chronic conditions in primary care settings: Critical review of concepts and 3 tools. Can. Fam. Physician.

[7] Jacobson A.M., Barofsky I., Cleary P., Rand L.L. (1988). Reliability and validity of a diabetes quality-of-life measure for the diabetes control and complications trial (DCCT). Diabetes Care.

[8] Vickery C.W., Blazeby J.M., Conroy T., Arraras J., Sezer O., Koller M., Rosemeyer D., Johnson C.D., Alderson D., EORTC Quality of Life Group (2001). Development of an EORTC disease-specific quality of life module for use in patients with gastric cancer. Eur. J. Cancer.

[9] Inanır I., Aydemir Ö., Gündüz K., Esen Danacı A., Türel A. (2006). Developing a quality of life instrument in patients with psoriasis: The Psoriasis Quality of Life Questionnaire (PQLQ). Int. J. Dermatol..

[10] Flanagan J.C. (1978). A research approach to improving our quality of life. Am. Psychol..

[11] Flanagan J.C. (1982). Measurement of the quality of life: Current state of the art. Arch. Phys. Med. Rehabil..

[12] WHOQOL Group (1998). WHO Quality of Life Scale (WHOQOL). Psychol. Med..

[13] Ware J.E., Sherbourne C.D. (1992). The MOS 36-item short-form health survey (SF-36): I. Conceptual framework and item selection. Med. Care.

[14] Bujang M.A., Lai W.H., Hon Y.K., Yap E.P.P., Tiong X.T., Ratnasingam S., Kim A.R.J., Husin M., Jee Y.Y.H., Ahmad N.F.D. (2023). Measuring population health and quality of life: Developing and testing of the significant quality of life measure. Heliyon.

[15] Bujang M.A. (2026). A Review of Generic Frameworks and Scales for Measuring Quality of Life and Well-Being: Toward Finding the Right Ruler. Behav. Sci..

[16] Bujang M.A., Husin M., Lai W.H., Hon Y.K., Tiong X.T., Kim A.R.J., Ahmad N.F.D., Yap E.P.P., Fong A.Y.Y. (2026). The revised version of significant quality of life measure (SigQOLM-36). BMC Res. Notes.

[17] Bujang M.A., Sa’at N., Bakar T.M.I.T.A. (2017). Determination of minimum sample size requirement for multiple linear regression and analysis of covariance based on experimental and non-experimental studies. Epidemiol. Biostat. Public Health.

[18] Levine T.R., Hullett C.R. (2002). Eta-squared, partial eta-squared, and misreporting of effect size in communication research. Hum. Commun. Res..

[19] Bujang M.A. (2025). The dilemma and wisdom in translating p values: A collaborative approach to strengthening scientific validity. BioMed Res. Int..

[20] Berghöfer A., Martin L., Hense S., Weinmann S., Roll S. (2020). Quality of life in patients with severe mental illness: A cross-sectional survey in an integrated outpatient health care model. Qual. Life Res..

[21] Pačarić S., Turk T., Erić I., Orkić Ž., Petek Erić A., Milostić-Srb A., Farčić N., Barać I., Nemčić A. (2020). Assessment of the quality of life in patients before and after coronary artery bypass grafting (CABG): A prospective study. Int. J. Environ. Res. Public Health.

[22] Rikos N., Kassotaki A., Frantzeskaki C., Fragiadaki M., Mpalaskas A., Vasilopoulos G., Linardakis M. (2023). Investigation of perception of quality of life and psychological burden of patients undergoing hemodialysis—Quality of life of hemodialysis patients. Nurs. Rep..

[23] Rosenberg S.M., Dominici L.S., Gelber S., Poorvu P.D., Ruddy K.J., Wong J.S., Tamimi R.M., Schapira L., Come S., Peppercorn J.M. (2020). Association of breast cancer surgery with quality of life and psychosocial well-being in young breast cancer survivors. JAMA Surg..

[24] Szlenk-Czyczerska E., Guzek M., Bielska D.E., Ławnik A., Polański P., Kurpas D. (2022). The analysis of the relationship between the quality of life level and expectations of patients with cardiovascular diseases under the home care of primary care nurses. Int. J. Environ. Res. Public Health.

[25] Bishop M. (2005). Quality of life and psychosocial adaptation to chronic illness and disability: Preliminary analysis of a conceptual and theoretical synthesis. Rehabil. Couns. Bull..

[26] Kalfas M., Chisari C., Windgassen S. (2022). Psychosocial factors associated with pain and health-related quality of life in endometriosis: A systematic review. Eur. J. Pain.

[27] Bujang M.A., Musa R., Liu W.J., Chew T.F., Lim C.T.S., Morad Z. (2015). Depressive disorders, anxiety and stress among patients with dialysis and the association with quality of life. Asian J. Psychiatry.

